# GNSS-R Altimetry Performance Analysis for the GEROS Experiment on Board the International Space Station

**DOI:** 10.3390/s17071583

**Published:** 2017-07-06

**Authors:** Adriano Camps, Hyuk Park, Ivan Sekulic, Juan Manuel Rius

**Affiliations:** 1 CommSensLab, Unidad de Excelencia María de Maeztu, Department of Signal Theory and Communications, Universitat Politècnica de Catalunya, E-08034 Barcelona, Spain; park.hyuk@tsc.upc.edu (H.P.); ivan.sekulic@tsc.upc.edu (I.S.); rius@tsc.upc.edu (J.M.R.); 2 Institut d’Estudis Espacial de Catalunya/Centre de Tecnologies Espacials-Universitat Politècnica de Catalunya, UPC Campus Nord, E-08034 Barcelona, Spain

**Keywords:** GNSS-R, altimetry, error budget, ionosphere, scintillations

## Abstract

The GEROS-ISS (GNSS rEflectometry, Radio Occultation and Scatterometry onboard International Space Station) is an innovative experiment for climate research, proposed in 2011 within a call of the European Space Agency (ESA). This proposal was the only one selected for further studies by ESA out of ~25 ones that were submitted. In this work, the instrument performance for the near-nadir altimetry (GNSS-R) mode is assessed, including the effects of multi-path in the ISS structure, the electromagnetic-bias, and the orbital height decay. In the absence of ionospheric scintillations, the altimetry rms error is <50 cm for a swath <~250 km and for U_10_ <10 m/s. If the transmitted power is 3 dB higher (likely to happen at beginning of life of the GNSS spacecrafts), mission requirements (rms error is <50 cm) are met for all ISS heights and for U_10_ up to 15 m/s. However, around 1.5 GHz, the ionosphere can induce significant fading, from 2 to >20 dB at equatorial regions, mainly after sunset, which will seriously degrade the altimetry and the scatterometry performances of the instrument.

## 1. Introduction

This manuscript presents an analysis of the altimetry performance of GEROS-ISS: a future space borne GNSS-R (Global Navigation Satellite System Reflectometry) experiment onboard the Columbus module of the International Space Station. GNSS-R is a relatively novel technique that uses navigation signals as signals of opportunity in a sort of bistatic radar. Scatterometry observations can be performed by comparing the power of the left hand circularly polarized direct signal, and the (mostly) right hand circularly polarized reflected one. Altimetry observations can be performed by comparing the differential time of arrival between the reflected and the direct signals. The precise measurement of this delay is affected by instrument errors, but by errors in the platform itself (varying orbital height and multipath), by ionospheric effects and surface scattering mechanisms. This work summarizes the results of the altimetry error budget carried out during one of the two Phase A studies of GEROS-ISS.

### 1.1. The Mission

The main goal of the GEROS-ISS (GNSS rEflectometry, Radio Occultation and Scatterometry onboard International Space Station) [1] is to demonstrate the capabilities of GNSS remote sensing to derive geophysical parameters of ocean, ice and land surfaces. “The main mission objectives of GEROS are (in order of priority):

- To measure and map altimetric sea surface height of the ocean with an accuracy of 30 cm or better (goal: 20 cm) using reflected GNSS signals to allow methodology demonstration, establishment of error budget and resolutions and comparison/synergy with results of satellite based nadir-pointing altimeters. This includes Precise Orbit Determination of the GEROS payload.
- To retrieve scalar ocean surface mean square slope (MSS), which is related to sea roughness, wind speed, with a GNSS spaceborne receiver to allow methodology testing, establishment of error budget and resolutions. MSS accuracy should be equivalent to a wind accuracy of 10% or 2 m/s whichever is greater. In addition, 2D MSS (directional MSS, related to wind direction) would be desirable.
- To assess the potential of GNSS scatterometry for land applications and in particular to develop products such as soil moisture, vegetation biomass, and mid-latitudes snow/ice properties and to further explore the potential of GNSS radio occultation data (vertical profiles of atmospheric bending angle, refractivity, temperature, pressure, humidity and electron density), particularly in the Tropics, to detect changes in atmospheric temperature and climate relevant parameters (e.g., tropopause height) and to provide additional information for the analysis of the reflectometry data from GEROS” (from [2]).

**Figure 1:**
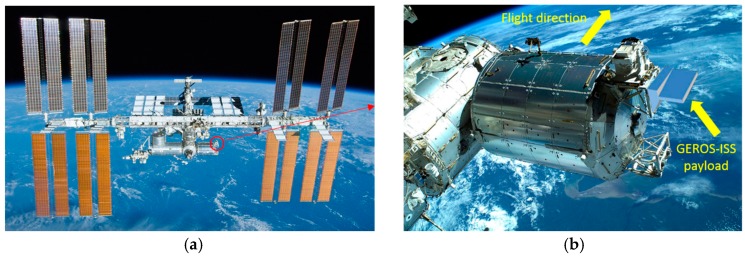
(**a**) View of the International Space Station, and (**b**) view of the Columbus module that will host GEROS-ISS payload in the upper deck.

The GEROS-ISS payload will be attached to the Upper Limb Balcony of the Columbus External Payload Facility part of the International Space Station as shown in Figure 1.

### 1.2. GEROS-ISS: The Payload

The GEROS-ISS payload [2] will collect reflected navigation signals (GPS and Galileo, and eventually Compass and Beidou as well) at left hand circular polarization (LHCP) and right hand circular polarization (RHCP), as well as radio occultation of navigation signals (with a minimum antenna gain =9 dBi) at both RHCP and LHCP. GEROS will be installed in the upper deck of the Columbus module, the European module on board the International Space Station.

**Figure 2:**
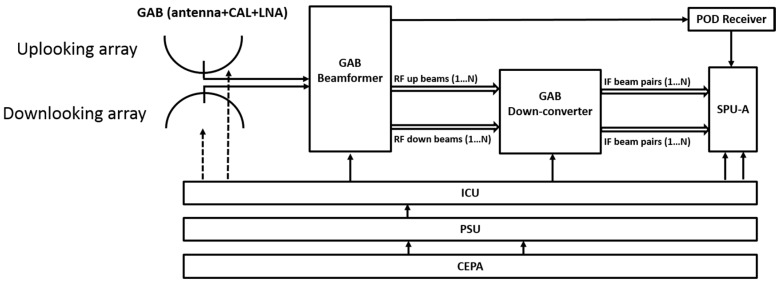
GEROS-ISS payload high level block diagram. Acronyms: GAB: GEROS Antenna Beamformer, ICU: Instrument Control Unit; PSU: Power Supply Unit; CEPA: Columbus External Payload Adapter; and SPU: Signal Processing Unit (SPU B removed during one of the Phase A studies).

The GEROS Antenna Beamformer (GAB, see Figure 2) antenna will consist of 2 dual-band multi-beam (4 beams min.) up- and down-looking arrays, mounted back-to-back, and pointing dynamically (from boresight to 77° out-of-boresight), in opposite directions towards the reflection points and their corresponding direct signals. The up- and down-looking antennas will be dual-polarization (RHCP and LHCP, respectively).

The GAB will receive and form beams with GPS and Galileo signals at L1/E1 and L5/E5 (including signals from its overlay GEO satellites), and Beidou and Compass signals at B1/C1 and B5/C5. GAB’s noise figure including antenna losses will be smaller than 3.5 dB.

The GAB will include dedicated beams for dual-frequency Precise Orbit Determination (POD), conveniently tapered to avoid multipath from nearby structures as much as possible. The calibration-low noise amplifier (CAL-LNA) front-end will be placed in between the up- and down-looking arrays, and will be capable of providing the following antenna outputs at all specified frequencies: RHCP_up_ and either LHCP_down_ or RHCP_down_, or both RHCP_down_ and LHCP_down_. Any signal could be routed to any receiver up to 16 times per second, to synthesize any polarization basis.

The main observables generated by the payload will be: (1) near nadir altimetry power waveforms of LHCP GNSS-R signals at the two specified frequency bands, (2) scatterometry, (3) land/cryosphere observables (RHCP and LHCP waveforms interlaced up to 16 times per second [2]), (4) carrier phase evolution between the direct RHCP and the reflected LHCP signals for grazing angle altimetry, and (5) Radio Occultations and Polarimetric Radio Occultations.

## 2. Methodology: Computation of the Altimetry Performance

### 2.1. Basic Instrument Performance

The estimation of the altimetry performance in terms of the delay lag *τ_p_* of the waveform can be estimated using the Cramér-Rao bound (CRB) (Equation (1)), which is expressed here in the time-domain and discrete form [3]:

(1) $$\sigma_{\tau_{p}}^{2}\geq \frac{1}{\sum_{k,l}{\overset{=}{C}}_{k,l}^{-1}s\prime \left(k-\tau_{p}\right)s\prime \left(l-\tau_{p}\right)}$$

where $\overset{=}{C}$ is the covariance matrix of the waveform s(τ), and s′ is the waveform’s derivative. In the case of white noise, uncorrelated from sample to sample, the covariance matrix is diagonal, with elements of noise power $\sigma_{n}^{2}$, and Equation (1) reduces to:

(2) $$\sigma_{\tau_{p}}^{2}\geq \frac{\sigma_{n}^{2}}{\sum_{l}{\left\{s\prime \left(l\right)\right\}}^{2}}$$

However, noise is not white for all waveform lags, there is some correlation between consecutive lags, and to some extent between consecutive waveforms (even though in space-borne applications it is negligible). Around the waveform peak, speckle noise dominates, degrading more the signal-to-noise ratio (SNR), and the overall performance. A simple formulation to account for the speckle noise and the noise correlation was originally derived in [4] and then used in [5,6]:

(3) $$\sigma_{\tau_{p}}^{2}\geq \frac{1}{SNR\cdot \sum_{l}\frac{{\left\{\bar{s}\prime \left(l\right)\right\}}^{2}}{R\left(l\right)}}$$

where $R\left(l\right)\hat{=}N_{i}/N_{i,eff}\left(l\right)$, where *N_i_* is the number of incoherent averages, and *N_i,eff_* (*l*) is the effective number of incoherent averages.

If R(l)=1, no speckle, just white thermal noise, the variance of the optimum achievable delay precision is given [7]:

(4) $$\sigma_{\tau_{p}}^{2}\geq \frac{1}{SNR\left(B\right)}\cdot \frac{1}{{\left(2\pi \beta \left(B\right)\right)}^{2}}$$

which depends on the *SNR* and *β* [Hz], the so-called rms or Gabor bandwidth:

(5) $$\beta^{2}=\frac{\int f^{2}{\left|S\left(f\right)\right|}^{2}df}{\int {\left|S\left(f\right)\right|}^{2}df}$$

being ${\left|S\left(f\right)\right|}^{2}$ the spectrum of the signal, including the effect of the frequency responses of the transmitter and receiver. In Equation (4), it has been made explicit that both the SNR and the Gabor bandwidth depend on the receivers’ bandwidth (B). Note that if R=1, Equations (3) and (4) are formally identical. This is an interesting result because usually one tends to think that the larger the (Gabor) bandwidth, the better the achievable ranging (delay) precision, but this is ONLY true at a given SNR. On the contrary, for any Gabor bandwidth, the same ranging (delay) precision can be achieved, provided that the SNR is high enough.

Another important issue is hidden in Equations (3) and (4): the Cramer-Rao bound provides the absolute minimum variance that can be achieved, but this is only true if the estimator—in this case the delay estimator—is an unbiased one, and in Section 2 the presence of this bias will be demonstrated.

Table 1 (from [6], adapted from [8]) summarizes the main parameters of the GPS and Galileo signals, and the optimum bandwidths and achievable precision heights at nadir for a SNR =20 dB, to make it independent of the antenna gain and transmitted power. Table 1 (from [6]) shows the achievable altimetry performance assuming the dual-frequency correction for the ionospheric delay. As it can be appreciated, the *σ*_h_ < 30 cm_rms_ requirements are only met at E1/E5 at nadir and at the swath edge for the typical and maximum transmitted powers, and at L1/L5 only at nadir and for the highest transmitted power. These results are revised in the later part of this paper, including an analysis of the electromagnetic bias, and other error sources missing in previous studies.

GEROS-ISS payload is very similar to the one in the PARIS in Orbit Demonstration [9], with which it shares many commonalities, although it will only implement the interferometric technique both for reflectometry and radio occultations. During the GEROS-ISS Phase A studies led by Astrium Defence and Space Spain (formerly EADS-CASA Espacio), the antenna size has proposed to be increased up to ~24 dB directivity @ L1/E1, and ~22 dB at L5/E5.

### 2.2. Non-Instrumental Error Sources in GNSS-R Altimetry

In addition to the geometric delay to be measured, there are several other contributions to the estimated average delay: the clock offsets of receiver and transmitter relative to GPS time scale, the electromagnetic (EM) bias, the tropospheric, and the ionospheric delays.

The EM bias basic is defined as the ratio of the average of the radar cross-section density $\left(\sigma^{0}\right)$ times the sea surface elevation  (ξ), divided by the average $\sigma^{0}\;$ [10]:

(6) $$\beta_{EM}=\frac{\langle \xi \sigma^{0}\rangle }{\langle \sigma^{0}\rangle }$$

Equation (6) takes into account that wave valleys are flatter and appear brighter in the scattered signal, than the wave crests. Therefore, the mean sea level appears to be at a lower height than it actually is.

The tropospheric delay has two contributions, the dry delay and the wet delay. The dry delay has an average value of 2.3 m, and a residual error of ~0.7 cm, while the tropospheric wet delay is highly variable, typically from 5 to 30 cm, and can be computed using atmospheric models or microwave radiometers, with a precision of ~1.1 cm.

The ionospheric delay is also highly variable, typically from 1 to 20 m, and it is estimated using dual-frequency observations with a precision of ~0.5 cm. However, the impact of ionospheric amplitude and phase scintillations in GNSS-R have not been assessed yet.

#### 2.2.1. Electromagnetic Bias

The evaluation of Equation (6) can be done analytically, but requires many assumptions and approximation in order to be mathematically treatable, and in the end, it requires a numerical integration. In [11], a technique was proposed to evaluate Equation (6) numerically, with a minimum number of approximations. To do that the surface wave height and $\sigma^{0}$ are required. The sea surface wave height and orientation of each facet are known, as they are the outputs of a temporal sea surface generator. The value of $\sigma^{0}\;$is computed using the Physical Optics under the Kirchhoff Approximation (KA-PO).

The main results are summarized in Table 2. Note that the EM bias increases with increasing incidence angle, and it can be as high as ~−25 cm, with an azimuthal signature up to 5 cm, _peak-to-peak_ for U_10_ = 15 m/s. EM bias must be corrected by means of look-up tables, wind speed and direction auxiliary data, but still if the wind speed has an uncertainty of ±2 m/s, the residual EM bias can be as high as ±5 cm.

#### 2.2.2. Impact of the Ionosphere

When an electromagnetic wave traverses the ionosphere, the time delay in excess of the propagation time in free space (*τ* [ns]) depends on the frequency (*f* [Hz]), and the total electron content (TEC) of along the slant propagation path (STEC [e^−^/m^2^]) as:

(7) $$\tau =134.5\cdot \frac{STEC}{f^{2}}$$

Then, ionospheric effects can be eliminated by combining the pseudo-range estimates at two different frequencies (e.g., L1/E1 and L5/E5) averaged over *N* (*N_typ_* = 3, [6,9]) consecutive observations:

(8) $${\hat{h}}_{15}=-\frac{1}{2}\cdot \frac{\rho_{1}+\rho_{5}}{2\cdot sin\theta_{e}}+\frac{1}{2}\cdot \left(f_{1}^{-2}-f_{5}^{-2}\right)\frac{{\langle I\prime \rangle }_{N}}{2\cdot sin\theta_{e}}$$

Assuming that the height error is different at both frequency bands (e.g., Table 1), the standard deviation of the resulting altimetry product is then given by [6]:

(9) $$\sigma_{{\hat{h}}_{15}}=\sqrt{1+\frac{1.26^{2}}{N}+\frac{2.26^{2}}{N}}\cdot \frac{\sqrt{\sigma_{h_{1}}^{2}+\sigma_{h_{5}}^{2}}}{2}$$

**Figure 3:**
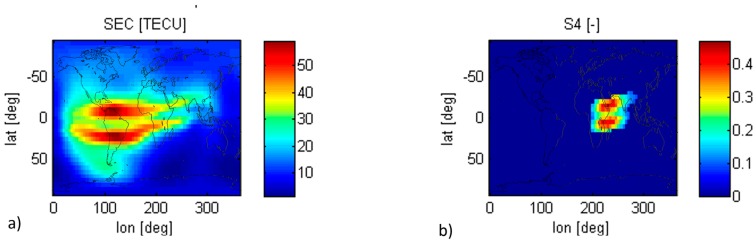
(**a**) Slant Electron Content maps in [TECU] computed with GISM for, and associated, (**b**) Scintillation Index S_4_ maps for paths for a receiver on ground. UT = 6 AM.

However, the accuracy of this correction used in navigation receivers, still needs to be validated, because the ISS height varies in time from 330 to 460 km, and it is in the middle of the ionosphere peak (maximum TEC). Figure 3a shows a global map of TEC computed using the Global Ionospheric Scintillation Model (GISM) [12] for 28 February 2015, at Universal Time UT = 6 AM. Note that maximum TEC content occurs around noon (local time), and around 20° North/South of the magnetic equator.

On the other hand, when an electromagnetic wave transverses the ionosphere, it suffers from intensity (*I*) and phase (*φ*) scintillations (rapid variations). The strength of the phase scintillations is characterized by *σ_φ_*, the standard deviation of the phase fluctuations, while the strength of the intensity scintillations is characterized by the scintillation index (*S*_4_) defined as:

(10) $$S_{4}=\sqrt{\frac{\langle I^{2}\rangle -{\langle I\rangle }^{2}}{{\langle I\rangle }^{2}}}$$

Figure 3b shows a global map of *S*_4_ computed using GISM for the same date and UT as in Figure 3a. There are two intense zones of scintillation, one at high latitudes and the other centered within ±20° of the magnetic equator, where depth of the scintillation fading ranges from 2 to more than 20 dB depending on the solar activity [12], and has time constants from 0.5 to 2 s. At mid latitudes scintillation occurs exceptionally, e.g., during geomagnetic storms. Scintillation is maximum after sunset (from ~19 to 24 h), and around the vernal and autumnal equinoxes, and scintillation events can last from 30 min to hours.

**Figure 4:**
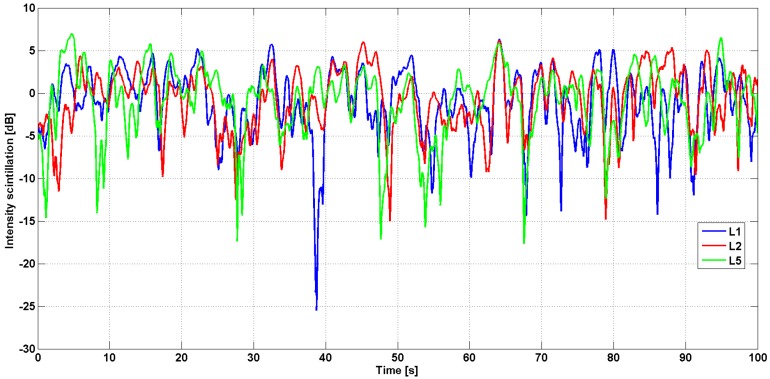
Simulated One-way ionosphere amplitude scintillations for S_4_= 0.8 and *σ_φ_* = 0.1 rad, at L1/E1 (blue), L2/E2 (red), and L5/E5 (red).

Finally, Figure 4 presents simulated amplitude scintillations at L1/E1, L2/E2 and L5/E5 bands computed for $S_{4}=0.8$ and *σ_φ_* = 0.1 rad. As it can be observed, large intensity fadings occur, and they are different at different frequency bands (i.e., 1575.42 MHz for L1/E1 at 1176.45 MHz for L5/E5a). They are closer among L2/E2 and L5/E5 because these frequencies are much closer. When the fading is large, the signal-to-noise ratio decreases, the range estimates get worse, and eventually, the signal may be totally lost, although often the SNR degradation is just a few dB. This effect cannot be calibrated using the up-looking antenna since the reflected signal passes through the ionosphere twice (completely in the down-welling path, and through most of it in the up-welling path depending on the orbital height), while the direct signal suffers much smaller ionospheric disturbances as it is only affected by the tenuous fraction of the ionosphere above the receiver.

In order to mitigate ionospheric effects, a Sun-synchronous orbit has to be selected, preferably a 6 a.m.–6 p.m. one, because it avoids passing through the ionosphere after sun set and midnight local time. If this is not possible, e.g., GEROS experiment on-board the ISS, a potential solution may consist of the calculation of a running average and a running standard deviation of consecutive observables (e.g., 10) and check if the expected standard deviation is consistent with the expected one, either from theory or from previous observations. If it is anomalously larger, this would be a clear indication of ionospheric scintillation (or of the presence of radio frequency interference) and therefore measurements could be flagged in real time.

### 2.3. Instrumental Error Sources in GNSS-R Altimetry

**Figure 5:**
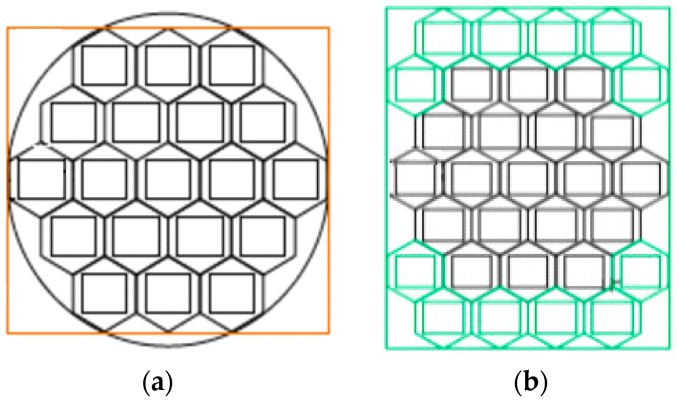
(**a**) Original antenna in PARIS IoD, and (**b**) antenna of GEROS ISS corresponding to the Phase A study led by Airbus Defense and Military.

**Figure 6:**
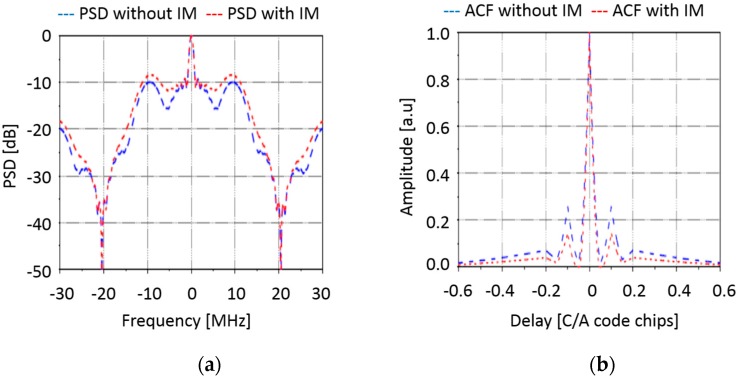
(**a**) Power Spectral Density (PSD), and (**b**) squared auto-correlation function (ACF) of the composite GPS L1 signal with and without considering the IM component (Adapted from [14]).

The procedure followed to compute the GEROS ISS experiment performance follows that in [6] for the PARIS In-orbit Demonstrator (IoD) Mission, with the following differences:

- Maximum and minimum ISS orbital heights considered to compute the maximum off-boresight angle for a given swath of 500 km (i.e., 250 km half swath in each side, nominal off-boresight angle = 35°).
- Antenna: instead of the hexagonal 19 element antenna array (Figure 5a) foreseen for PARIS IoD [6], a rectangular 31 element antenna array (Figure 5b) was selected for GEROS-ISS, since larger apertures fit in the available space in the upper deck of the Columbus module. This translates into an increased directivity and increased signal-to-noise ratio. The array topology and the numerically computed elementary antenna patterns have been used to estimate the directivities for the up and down-looking antennas, at boresight and at 35° or the corresponding angle to the maximum off-boresight angle for each orbital height, and swath, and for the lower (L5/E5) and upper (L1/E1) bands. Array parameters are summarized in Table 3.
- Receivers noise figure is NF_nom_ = 3.5 dB.
- Receivers bandwidth is B = 40 MHz.
- Dwell line is 100 km, unless otherwise specified. Results for a 1 ms coherent integration, and 1 s integration (incoherent averaging) time are also provided.
- All other instrumental error sources, etc. as in [6].
- Inter-modulation signal power is taken into account. The inter-Modulation (IM) components are extra signal components transmitted by the GPS navigation satellites to keep the power envelope of the composite signals constant, so as to improve the performance of the solid state power amplifiers (SSPAs). The IM signals do not transmit navigation information, so they have no impact in conventional GNSS-R (cGNSS-R) or reconstructed-code GNSS-R (rGNSS-R) [13], but they do in the interferometric GNSS-R (iGNSS-R), because this later technique cross-correlates the whole signal. The IM signal accounts for a 25% of the total transmitted power, so the SNR is increased by 4/3 (1.25 dB). Figure 6a,b show the power spectral density (PSD), and the squared auto-correlation function (ACF) of the composite GPS L1 signal with (red) and without (blue) considering the IM component. As it can be appreciated, not only the power is higher, but it is more concentrated towards the higher frequencies, which increases the Gabor bandwidth (Equation (5)), and the achievable altimetry precision.

Since both, single frequency or dual-frequency (ionosphere corrected) altimetry estimates depend on the bandwidth and the SNR (Equation (4)), for a given receiver’s bandwidth, the impact of any error source reduces to the calculation of its impact on the signal or the noise powers, and therefore, the two most important parameters are the receivers’ noise figure and the antenna directivity.

### 2.4. Platform Error Sources in GNSS-R Altimetry

#### 2.4.1. Impact of ISS Orbital Height Decay

**Figure 7:**
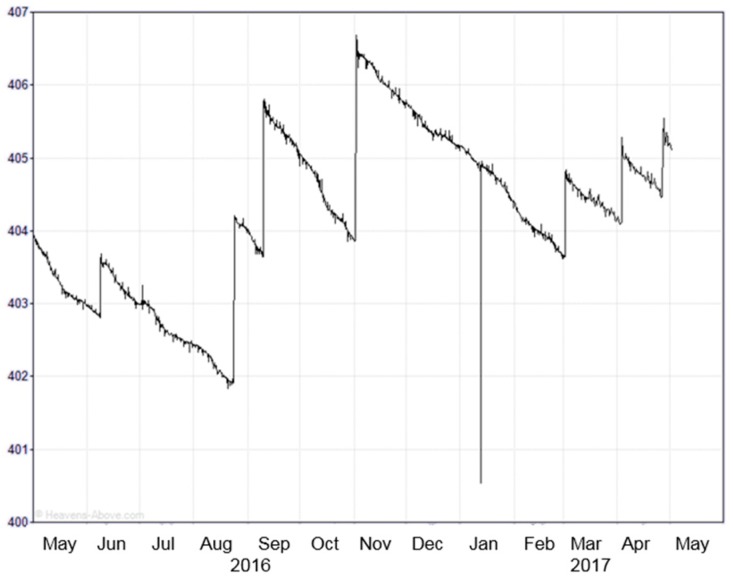
Evolution of the ISS orbital height during one year: periodic impulses to increase ISSS height, and gradual fall between them are clearly visible. Gradual fall is caused by the atmospheric drag. Non-constant descent rate is due to changes in the upper atmosphere density, mainly due to solar activity [http://www.heavens-above.com/IssHeight.aspx].

The decay rate of the ISS can vary significantly along time, with sharp vertical transitions corresponding to orbit re-boosts to move the ISS to higher altitudes (e.g., Figure 7, from http://www.heavens-above.com/IssHeight.aspx). During solar maximum conditions, the ISS loses 400 m of altitude per day (4.63 mm/s), while during sunspot minimum conditions the rate is only 80 m per day (0.92 mm/s). These values will have to be taken into account during the long incoherent integration times foreseen in GEROS-ISS (~13.6–14 s for a dwell line of 100 km), and when computing the statistics of GEROS-ISS estimated height at different time instants. In practical terms, from an implementation point of view of the receiver it will require to split the incoherent averaging period into shorter integration times, and realign (re-track) the waveforms, before performing the second incoherent averaging [15].

#### 2.4.2. Impact of ISS Shadowing and Multiple Scattering

**Figure 8:**
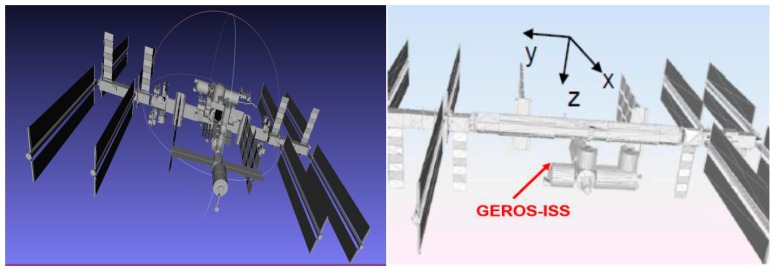
Simplified ISS model and reference frame used.

The ISS has a number of advantages for experimental payload installation, power availability etc., but it also has some drawbacks that have to be taken into account in a payload like this one. The potential shadowing and multiple scattering have to carefully analyzed because they can seriously distort the observations. In order to do that, a full model of the ISS has been used [16], which consists of 551,748 triangles. Since this was a high number for a detailed electromagnetic simulation, a simplified model eliminating hidden parts, with just 11,620 triangles, more amenable for the analysis was used instead. Large flat areas were grouped, and hidden parts removed, as shown in Figure 8. The software package used is newFASANT [17], a commercial one, with custom made tuning for improved computational speed in our particular scenario. Multiple reflections over flat and curved surfaces up to third order (two reflections) were found. Using the reciprocity theorem, calculations have assumed a transmitting isotropic antenna located in the position of the receiving antenna, and for each direction in the space (far-field), the different contributions (direct, the second order, and third order reflections) are computed.

**Figure 9:**
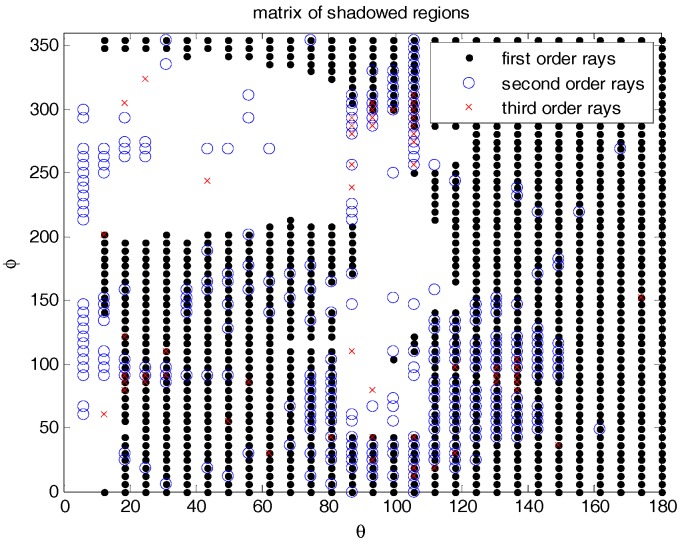
Graphical representation of the directions of arrival of direct signal or first order rays (no reflections) (**·**), second order rays (one reflection) (**o**), and third order rays (two-reflections) (**x**). Empty areas correspond to shadowed directions blocked by the ISS structure.

**Figure 10:**
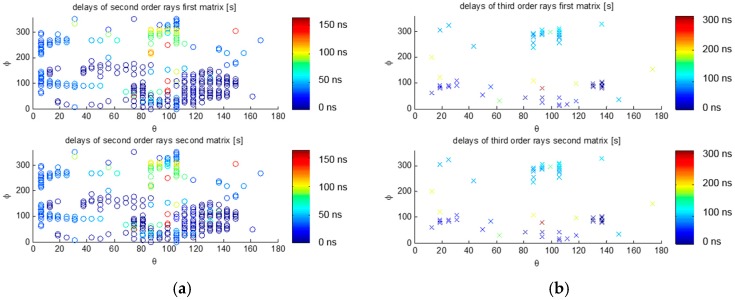
(**a**) Delays for the second order rays, and (**b**) for the third order rays. Direction of Arrival (*θ*, *φ*) in the antenna far field. Co-polar antenna pattern used for the direct and third order rays, cross-polar pattern used for the second order rays. Top: first matrix and bottom: second matrix, refer to the fact that there are second order and third order rays that have the same far field direction, but enter through different directions in the antenna pattern reference frame. Color code corresponds to the delay in ns.

Simulation results are shown in Figure 9 and Figure 10, where the notations of dot, circle o, and cross x indicate the directions of arrival of the direct signal, the second, and third order reflections, respectively (i.e., arrive to antenna after one, or two reflections). Empty areas correspond to shadowed directions blocked by the ISS structure. Note that two of the latest second order reflections occur at delays of ~150 ns and (*θ*, *φ*) ≈ (100°, ±90°), from the closest solar panels. The latest third order reflection occurs at a delay of ~300 ns and (*θ*, *φ*) ≈ (100°, 90°), again from the closest solar panel. The impact in the waveforms (or delay Doppler maps) is tricky to imagine intuitively, because of the different amplitude (in case of diffraction), and phases of each reflection, and because the scattered fields’ polarization changes, so it is collected through the co- or cross-polar antenna patterns.

**Figure 11:**
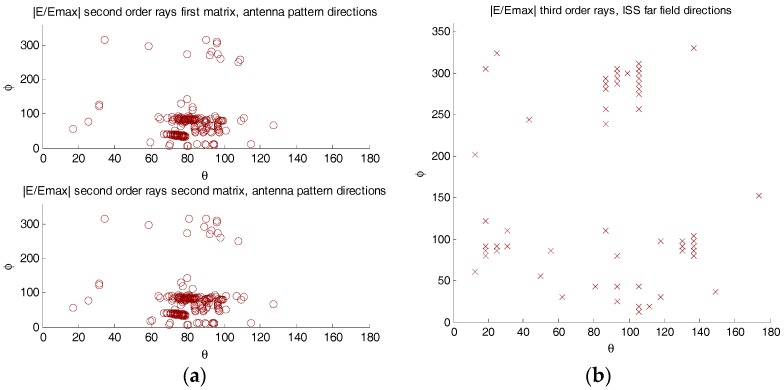
(**a**) Second order arriving direction to GEROS-ISS in the antenna reference frame, and (**b**) third order rays arriving direction to GEROS-ISS in the ISS far field reference frame. Top: first matrix and bottom: second matrix, refer to the fact that there are second order and third order rays that have the same far field direction, but enter through different directions in the antenna pattern reference frame. Color code corresponds to the delay in ns.

The total electric field at a given time instant is the sum of the first, second, and third order rays that reach the antenna with different time delays, and through different directions (and polarizations) of the antenna pattern. Regarding the polarization, the first order rays (direct ones) can be considered co-polar (LHCP for the down-looking antenna, and RHCP for the up-looking antenna). For the reflected signal, it is assumed that the each reflection over a metallic surface reverses the polarization perfectly, while amplitude of the reflection coefficient is $\left|\Gamma_{p}\right|=1$. Correspondingly, the second order rays are weighted by the cross-polar antenna pattern (RHCP for the down-looking antenna, and LHCP for the up-looking antenna), and the third rays are weighted by the co-polar patterns, as the first order rays.

(11a) $${\vec{E}}_{up}={\vec{E}}_{dir}\left\{F_{RH}\left(\theta_{1}^{dir},\phi_{1}^{dir}\right)+F_{LH}\left(\theta_{2}^{dir},\phi_{2}^{dir}\right)e^{-j\omega \tau_{2}^{dir}}+F_{RH}\left(\theta_{3}^{dir},\phi_{3}^{dir}\right)e^{-j\omega \tau_{3}^{dir}}\right\}+\;{\vec{E}}_{scat}\left\{F_{RH}\left(\theta_{2}^{scat},\phi_{2}^{scat}\right)e^{-j\omega \tau_{2}^{scat}}+F_{LH}\left(\theta_{3}^{scat},\phi_{3}^{scat}\right)e^{-j\omega \tau_{3}^{scat}}\right\},$$

(11b) $${\vec{E}}_{dn}={\vec{E}}_{scat}\{F_{LH}\left(\theta_{1}^{scat},\phi_{1}^{scat}\right)+F_{RH}\left(\theta_{2}^{scat},\phi_{2}^{scat}\right)e^{-j\omega \tau_{2}^{scat}}+F_{LH}\left(\theta_{3}^{scat},\phi_{3}^{scat}\right)e^{-j\omega \tau_{3}^{scat}}\}\;+{\vec{E}}_{dir}\left\{F_{LH}\left(\theta_{2}^{dir},\phi_{2}^{dir}\right)e^{-j\omega \tau_{2}^{dir}}+F_{RH}\left(\theta_{3}^{dir},\phi_{3}^{dir}\right)e^{-j\omega \tau_{3}^{dir}}\right\}\;,$$

where ${\vec{E}}_{up}$ and ${\vec{E}}_{dn}$ are the electric fields received by the up- and down-looking antenna, $F_{RH}\left(\theta_{n},\phi_{n}\right)$ is the RHCP antenna pattern (amplitude and phase), for the *n*^th^ order ray coming from the $\left(\theta_{n}^{dir},\phi_{n}^{dir}\right)$, and *τ*_n_ is the delay of the *n*^th^ order ray relative to the first order one. Figure 11 shows the directions of arrival to the antenna in the antenna reference frame.

Taking into account Equation (11a,b), the cross-correlation of the signals from up-looking and down-looking antennas can be computed ($\langle {\vec{E}}_{up}\cdot {\vec{E}}_{dn}^{*}\rangle$). An efficient way to compute the final GNSS-R observable consists of the computation of the convolution of the multi-path free waveforms ($W_{1}\left(\tau \right)$), and the impulse response that models the multiple reflections ($I_{m}\left(\tau \right)$):

(12) $$W_{f}\left(\tau \right)={W_{1}\left(\tau \right)|}_{\left(\theta_{1}^{dir},\;\phi_{1}^{dir};\;\theta_{1}^{scat},\;\phi_{1}^{scat}\right)}*{I_{m}\left(\tau \right)|}_{\left(\theta_{1}^{dir},\;\phi_{1}^{dir};\;\theta_{1}^{scat},\;\phi_{1}^{scat}\right)}$$

As seen in Equation (12), the multiple reflected rays contribute with additional replicas of the original waveform delayed and weighted by the corresponding antenna pattern of the direction of arrival in the antenna reference frame. Doppler effects can be neglected because of the short distances involved, and the static geometry of the ISS itself.

**Figure 12:**
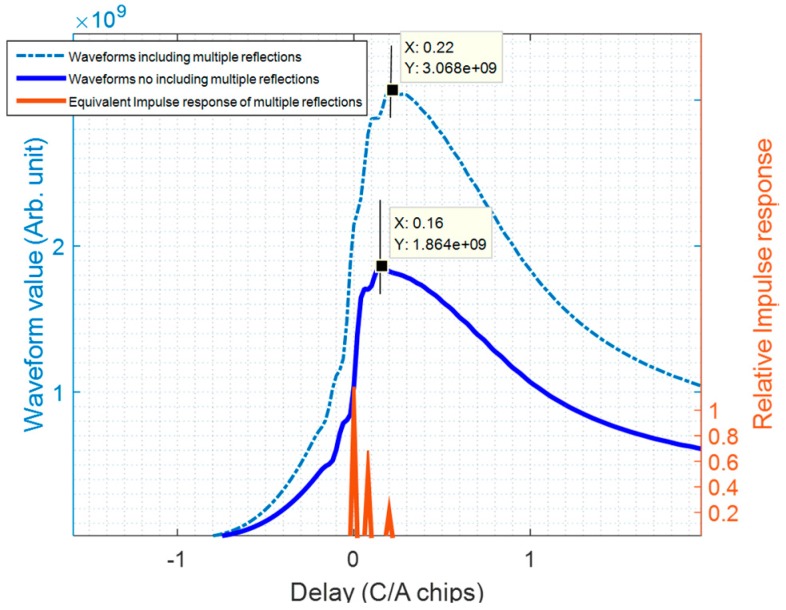
Example of the impact of multipath in the ISS structure.

For illustration purposes, assuming that $(\theta_{1}^{dir},\;\phi_{1}^{dir})=\left(124{}^{\circ},\;80{}^{\circ}\right)$ for the direct signal, and $(\theta_{1}^{scat},\;\phi_{1}^{scat})=\left(56{}^{\circ},\;84{}^{\circ}\right)$ for the far-field direction of the scattered signals. In this case, there are first and third order reflections from the scattered signals, and first and second order reflections from the direct signals. For the scattered signal, the direction of arrival of the third order reflection is $(\theta_{3}^{scat},\;\phi_{3}^{scat})=\left(76{}^{\circ},\;38{}^{\circ}\right)$, and the delay $\tau_{3}^{\mathrm{scat}}=0.188\;\mu s$ (56 m = 0.19 coarse/acquisition chips or C/A chips). For the direct signal, the direction of arrival of the second order reflection is $(\theta_{2}^{dir},\;\phi_{2}^{dir})=\left(79{}^{\circ},\;36{}^{\circ}\right)$ and the delay $\tau_{2}^{\mathrm{dir}}=0.188\;\mu s$ (24 m 0.08 C/A chips). Note that $\theta_{2}^{dir}<90{}^{\circ}$, i.e., the second order rays (single reflected) of the direct signal can be collected via down-looking antenna. Taking into account this multiple reflection, the simulation results are show in Figure 12. The waveform affected by multipath (light blue dash-dot line) $W_{f}\left(\tau \right)\;$is calculated as the convolution of the waveform $W_{1}\left(\tau \right)$ (dark blue solid line) and $I_{m}\left(\tau \right)$ (red peaks). Because of the multipath, the waveform peak increases by a factor of 1.7, and it is delayed by 0.06 C/A chips (=18 m), which is not negligible at all for altimetry purposes. The altimetric delay is found by the peak of the waveform derivative, and not by the waveform peak itself.

Despite this, multi-path can be mitigated by computing the waveforms not as the incoherent average of the individual coherent waveforms, but as the variance of the measured coherent waveforms instead of performing the incoherent average, as demonstrated in [18].

## 3. Results: Predicted GNSS-R Altimetry Performance

### 3.1. GNSS-R Altimetry Performance and Trade-off between Techniques

**Figure 13:**
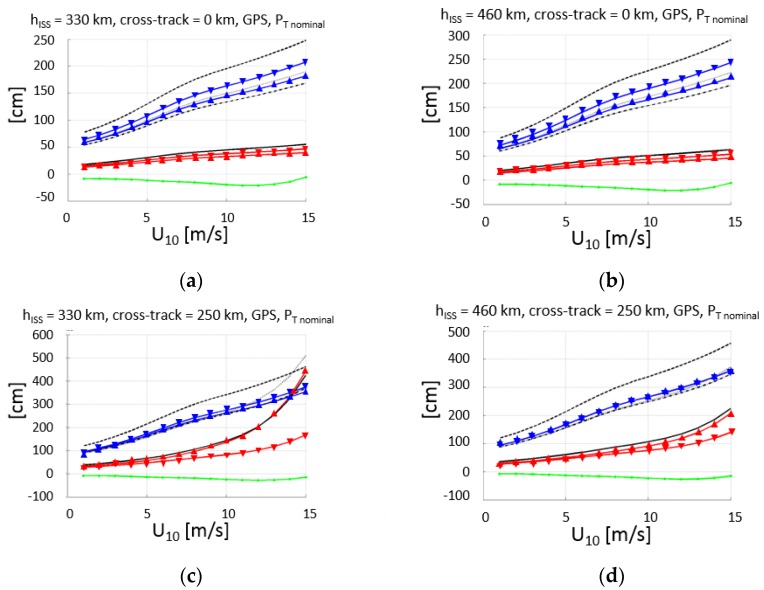
GPS constellation (nominal transmitted power [19], T_i_ = 1 s): predicted GEROS-ISS altimetry rms error (black), rms error at L1 (red) and L5 (blue), and total bias: electromagnetic bias and waveform bias (green), for h_ISS_ = 330 km (**a**) and (**c**) and 460 km (**b**) and (**d**), and for cross-track distance 0 km (**a**) and (**b**) and 250 km (**c**) and (**d**). Code: -Δ- σ_iF1_, -∇- σ_iF5_, -Δ- σ_cF1_, -∇- σ_cF5_, — σ_iF1+iF5_, - - - σ_cF1+cF5_, ····· σ_iF1+cF5_, -·-·- σ_cF1+iF5_, — bias_EM+WF._

**Figure 14:**
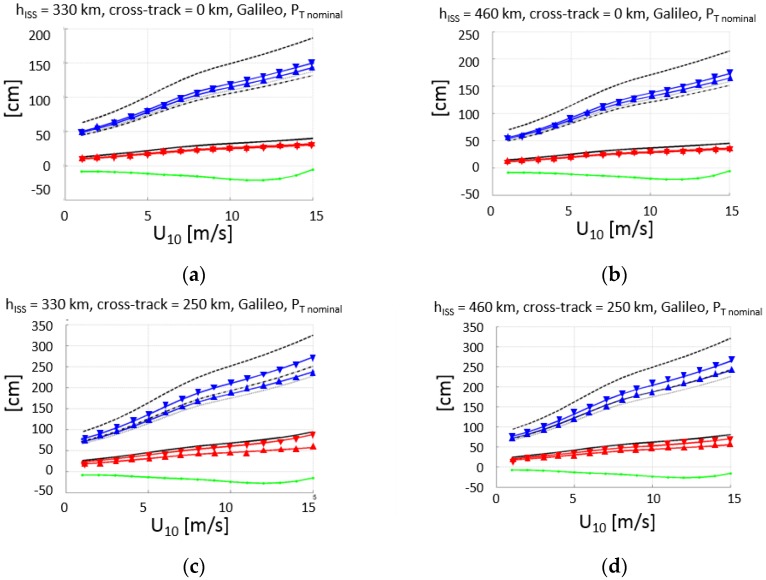
Galileo constellation (nominal transmitted power [20], T_i_ = 1 s): predicted GEROS-ISS altimetry rms error (black), rms error at L1 (red) and L5 (blue), and total bias: electromagnetic bias and waveform bias (green), for h_ISS_ = 330 km (**a**) and (**c**) and 460 km (**b**) and (**d**), and for cross-track distance 0 km (**a**) and (**b**) and 250 km (**c**) and (**d**). Code: -Δ- σ_iF1_, -∇- σ_iF5_, -Δ- σ_cF1_, -∇- σ_cF5_, — σ_iF1+iF5_, - - - σ_cF1+cF5_, ····· σ_iF1+cF5_, -·-·- σ_cF1+iF5_, — bias_EM+WF_.

Following the same procedure as in [6], and taking into account the effects discussed in Section 2.4.1 and Section 2.4.2, properly compensated for, the altimetry performance is computed for the nominal transmitted powers of the GPS (Figure 13) and Galileo (Figure 14) constellations, for the minimum (330 km) and maximum (460 km) ISS orbital heights, which require the maximum and minimum antenna off-boresight angles to satisfy the ±250 km swath requirement.

The altimetry performance is computed first individually for each band (F1 = L1 or E1, F5 = L5 or E5a), using either the conventional or the interferometric GNSS-R techniques (cGNSS-R or iGNSS-R). Then, the performance of the dual-frequency combination of these observables to compensate for the ionospheric delay (Equation (8)) is computed, that is, using either cGNSS-R or iGNSS-R, at either F1 or F5.

Table 4 summarizes the results in a numerical format. In this table, the results for the minimum and maximum transmitted powers by the space vehicles of the GPS and Galileo GNSS constellations are included.

It is worth to note that for the minimum transmitted powers, in all cases, except for Galileo at nadir, the performance of the iGNSS-R technique quickly degrades because of the marginal SNR, while cGNSS-R exhibits a more graceful degradation in terms of range estimation performance. At nadir, the wind speed at which the performance of iGNSS-R and cGNSS-R decreases with increasing height, while at the swath edge it decreases with increasing local incidence angle (e.g., lower height). These results can be used as inputs for assimilation studies to assess the scientific impact of the GEROS-ISS observations.

Assuming a 1 s incoherent integration time, for a nominal instrument with errors, the altimetric rms error is smaller than 70–90 cm at nadir (38–43 cm if the transmitted power is increased by 3 dB), and smaller than 11–17 m at the swath edge (250 km) for U_10_ < 8–9 m/s (71–76 cm if the transmitted power is increased by 3 dB), and for any orbital height h_ISS_ = 330–460 km.

Finally, the altimetry performance for different combinations of GNSS-R techniques (iGNSS-R and cGNSS-R) is discussed. In particular, the use of iGNSS-R in both bands, and the use of iGNSS-R at F1 and cGNSS-R at F5, which will make it more robust in front of the severe RFI that it is likely to appear due to DME (Distance Measuring Equipment) and TACAN (TACtical Air Navigation system) systems [21]. It is worth noting that the performance degradation as compared to the use of cGNSS-R is typically a factor between 3 and 4, except for the lowest SNR, when the performance is basically the same (GPS, U_10_ = 15 m/s).

### 3.2. GNSS-R Altimetry Performance Sensitivity to Errors

**Figure 15:**
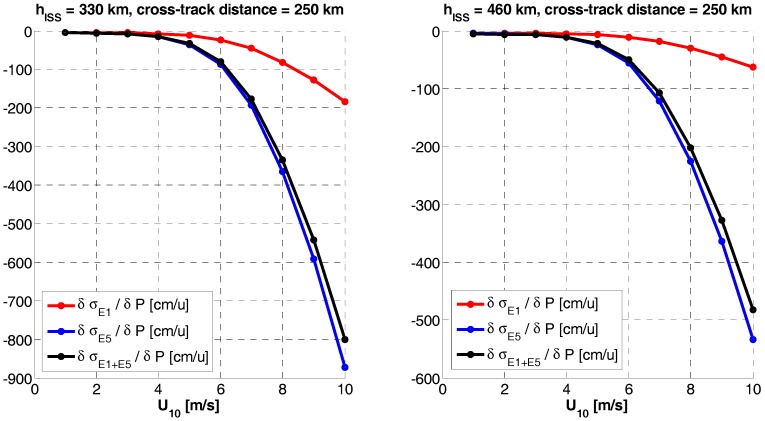
RMS height error sensitivity to antenna directivity (P) at nominal values as given by Table 3, u = [dB].

**Figure 16:**
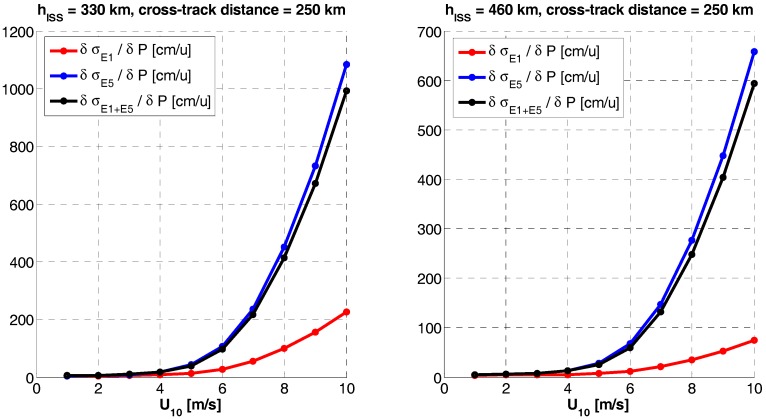
RMS height error sensitivity to receivers noise figure [dB]; errors (P) at nominal values (NF_nom_ = 3.5 dB), u = [dB].

Since both, single frequency or dual-frequency altimetry estimates depend on the bandwidth and the SNR, the sensitivity to any error source computed as the derivative of the rms altimetry error at a given frequency band ($\sigma_{f}$) with respect to a given parameter (*P*), $\partial \sigma_{f}/\partial P$, reduces to the calculation of the sensitivity to the signal and the noise powers, whose main drivers are the antenna directivity and the receivers’ noise figure, respectively. Since the sensitivities exhibit a highly non-linear behavior, it is not possible to provide a single numerical value. Therefore, the sensitivities to the antenna pattern directivity (Figure 15) and the receivers’ noise figure (Figure 16) are presented here. Please note that the parameters presented are the derivatives of the RMS error, so when it is negative, it means that the error decreases, and the other way around. It is clear the significant degradation (increase) of the RMSE, especially at the swath edges, because of the antenna directivity drop.

**Figure 17:**
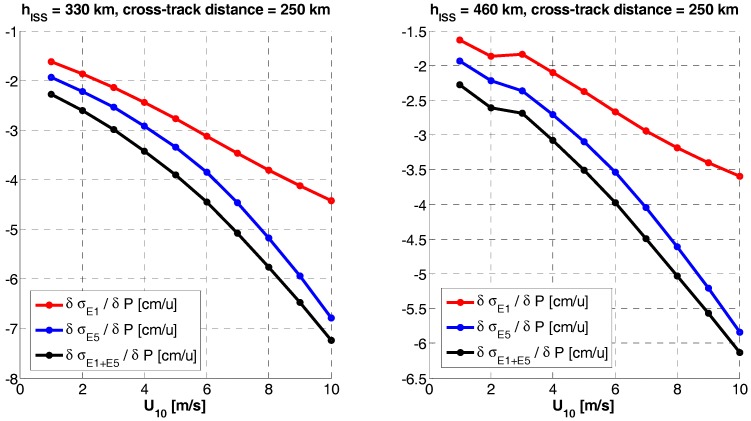
RMS height error sensitivity to antenna directivity (P) at nominal values +3 dB, u = [dB].

If the directivity increases, so does the *SNR*, then the sensitivity to errors is much smaller. A last comment is illustrated in Figure 17. Comparing it with Figure 15, it can be seen that if the transmitted power is just 3 dB more, then the sensitivity to the directivity dramatically decreases, since the SNR for the iGNSS-R does not increase any further.

Other influencing factors are the antenna losses, the beamformer amplitude errors, the array phase center, the number of bits in the phase shifter, the group delay error, the bandwidth rms mismatch, the number of bits of the correlator, the Delay Refresh Rate Update (DeRRU), and the Doppler Refresh Rate Update (DoRRU). The first three have an evident impact in the *SNR* before correlation. The others as well, but their impact is much more moderate, as compared to the antenna directivity and receivers’ noise figure.

## 4. Discussion

This work has evaluated the altimetry performance of the GEROS experiment on board the International Space Station. It is the natural continuation of the analysis carried out in [6], including:

(1) the use of the Cramer-Rao bound has been used to assess the performance of an ideal instrument performance, including now more directive antennas,
(2) ionospheric effects,
(3) the ISS orbit height decay rate, and
(4) multi-path propagation due to reflections in the ISS structure, including diffraction in curved surfaces. It has been shown that there are up to 3rd order rays (2 reflections), with delays spanning up to ~150 ns and ~300 ns for the 2nd and 3rd order rays. These coherent reflections can be mitigated as suggested in [18], but it will have an impact of the scatterometry observables over land and the cryosphere.

In addition, the four possible combinations to get the dual-frequency observables needed to correct for ionospheric effects has been assessed quantitatively, concluding that, despite the cGNSS-R has a more graceful degradation because of the higher SNR, the iGNSS-R performs better for the antenna patterns considered. However, the directivity values are in the border line, and a small decrease in the directivity value, translates into a quick degradation of the performances. Assuming 1 s incoherent integration time, for a nominal instrument with errors, the altimetric rms error is smaller than 70–90 cm at nadir, and smaller than 11–17 m at the swath edge (250 km) for U_10_ <~9 m/s, and for any orbital height h_ISS_ = 330–460 km.

## 5. Conclusions

Space borne GNSS-R altimetry from the ISS is feasible with a RMS error smaller than 30 cm_rms_ within the whole swath (*θ_i_* ≤ 35°) using Galileo signals (with GPS as well, but errors are beyond the GEROS-ISS requirements), when the space vehicles transmit at least their nominal power, and when dual-frequency observations are used in the ionospheric-free combination to cancel ionospheric effects (background TEC and refractive scintillations). Diffractive scintillations cannot be compensated for, but since they occur mostly after sunset and before midnight, either the measurements are discarded (or not even acquired), or a Sun-synchronous orbit with 6 a.m. or 6 p.m. LTAN (local time of the ascending node) can be selected.

Multiple scattering from the ISS structure has revealed that the strength of the received signals is affected (important for scatterometry), that the waveform peak is shifted by ~0.06 C/A chips = 18 m (important for altimetry), that there is a region where signals are blocked by the ISS itself, then, 4) that there are other regions in the space where two or up to three “rays” may reach the antenna arrays. In any case, multipath can be compensated for by computing the variance of the intensity waveforms, instead of the incoherent averaging.

Finally, the Sensitivity of the GNSS-R altimetry performance to instrumental errors has been computed, showing the criticality of the antenna pattern directivity, and the importance of keeping low noise figures in the receiver’s front-ends.

## Figures and Tables

**Table 1:** Altimetry rms error estimated by combining L1/E1 and L5/E5 bands to correct for the ionospheric delay at nadir and at an incidence angle of 35° (swath edge) (modified from [6]). LB = 1186.6 MHz and LH = 1575.42 MHz. Requirement is for *σ*_h_ < 30 cm_rms_ over 100 km dwell line.

Altimetry Precision [cm_rms_]		σ_h_ @ θ_i_ = 0°			σ_h_ @ θ_i_ = 35°		
		T_coh_ = 1 ms, N_i_ = 14,500			T_coh_ = 1 ms, N_i_ = 14,500		
		P_T,min_	P_T,typ_	P_T,max_	P_T,min_	P_T,typ_	P_T,max_
Level-1	L5	49.8	29.7	15.5	95.3	56.0	28.5
Lower band (L5 + E5)	E5	10.1	8.3	7.2	19.1	15.5	13.3
Level-1	L1	22.5	16.4	12.9	51.2	37.2	27.6
Higher band (L1 + E1)	E1	15.3	12.8	11.3	33.6	26.6	22.4
Level-2	L1&L5	49.2	30.5	18.1	97.4	60.5	35.7
(LB + HB + iono correct.)	E1&E5	16.5	13.7	12.3	34.8	27.7	23.4

**Table 2:** Average EM bias ${\bar{\beta }}_{EM}$ [cm] for incidence/scattering angles *θ*_i_ = *θ*_s_ = 0°, 25° and 45°, for wind direction *ϕ* = 45°, and U_10_ = 5 m/s, 10 m/s and 15 m/s.

U_10_	*θ_i,s_* = 0°	*θ_i,s_* = 25°	*θ_i,s_* = 45°
5 m/s	−3.89	−3.72	−4.96
10 m/s	−8.37	−9.73	−13.8
15 m/s	−13.35	−17.2	−24.1

**Table 3:** Average antenna array parameters used in the estimation of GEROS-ISS experiment performance.

	Upper Band (L1/E1)		Lower Band (L5/E5)	
	Boresight	35°	Boresight	35°
D_up-looking_	24.72 dB	20.02 dB	22.42 dB	19.62 dB
D_down-looking_	24.72 dB	22.12 dB	22.42 dB	20.82 dB

**Table 4:** GEROS-ISS Performance Summary [cm_rms_]: GPS and Galileo constellations; minimum, nominal and maximum transmitted power [19,20], U_10_ = 1–15 m/s, T_i_ = 1 s, four types of dual-frequency observables, and h_ISS_ = 330 km and 460 km, and for cross-track distances of 0 and 250 km.

				h_ISS_ = 330 km				h_ISS_ = 460 km			
GNSS	Cross Track	P_T_	U_10_ [m/s]	iF_1_-iF_5_	cF_1_-cF_5_	iF_1_-cF_5_	cF_1_-iF_5_	iF_1_-iF_5_	cF_1_-cF_5_	iF_1_-cF_5_	cF_1_-iF_5_
GPS	0 km	min	1	37	124	100	82	42	142	115	94
			15	541	505	428	604	1502	649	566	1535
		nom	1	18	77	58	53	20	87	66	60
			15	55	248	190	168	63	289	222	196
		max	1	10	53	37	39	11	58	41	44
			15	31	148	102	112	35	170	117	129
	250 km	min	1	99	202	165	153	84	201	161	146
			15	410	1446	249	326	195	1442	64	184
		nom	1	38	120	88	90	35	119	88	87
			15	425	462	508	368	225	456	372	346
		max	1	20	78	52	62	19	78	52	61
			15	64	254	161	207	58	251	161	201
Galileo	0 km	min	1	23	90	67	65	26	102	76	73
			15	71	306	227	217	85	363	270	257
		nom	1	13	62	46	44	14	69	51	49
			15	40	186	137	132	45	215	158	152
		max	1	6	41	30	29	7	45	33	32
			15	20	103	76	73	23	117	86	83
	250 km	min	1	52	144	105	112	47	143	104	109
			15	7763	647	7747	810	2439	632	2432	658
		nom	1	26	94	67	72	24	93	66	70
			15	95	325	228	250	81	321	225	242
		max	1	12	58	40	43	11	57	40	42
			15	37	167	115	127	35	166	116	124
